# KH176 under development for rare mitochondrial disease: a first in man randomized controlled clinical trial in healthy male volunteers

**DOI:** 10.1186/s13023-017-0715-0

**Published:** 2017-10-16

**Authors:** Saskia Koene, Edwin Spaans, Luc Van Bortel, Griet Van Lancker, Brant Delafontaine, Fabio Badilini, Julien Beyrath, Jan Smeitink

**Affiliations:** 1 Radboud Center for Mitochondrial Medicine (RCMM) at the Department of Pediatrics, Radboud university medical center, Geert Grooteplein Zuid 10, PO BOX 9101, 6500 HB Nijmegen, The Netherlands; 2 Khondrion BV, Philips van Leydenlaan 15 (427), 6525 EX Nijmegen, The Netherlands; 3 Drug Research Unit Ghent, Ghent University Hospital, De Pintelaan 185, 9000 Ghent, Belgium; 4 Analyzing Medical Parameters for Solutions (AMPS), New York, USA

**Keywords:** Randomized controlled trial, Mitochondrial medicine, Clinical trial phase 1, Mitochondrial disorder, Orphan drugs, Rare disease, KH176, Redox, Pharmacokinetics, Safety

## Abstract

**Background:**

Mitochondrial disorders are a clinically, biochemically and genetically heterogeneous group of multi-system diseases, with an unmet medical need for treatment. KH176 is an orally bio-available small molecule under development for the treatment of mitochondrial(−related) diseases. The compound is a member of a new class of drugs, acting as a potent intracellular redox-modulating agent essential for the control of oxidative and redox pathologies. The aim of this randomized, placebo controlled, double-blinded phase 1 study was to test safety, tolerability and pharmacokinetics of single and multiple doses of KH176 in healthy male volunteers. Putative effects on redox related biomarkers were explored.

**Results:**

KH176 was well tolerated up to and including a single dose of 800 mg and multiple doses of 400 mg b.i.d. for 7 Days. However, when the QT interval was corrected for heart rate, administration of single doses of 800 and 2000 mg and at a multiple dose of 400 mg KH176 had marked effects. Post-hoc analysis of the ECGs showed clear changes in cardiac electrophysiology at single doses of 800 and 2000 mg and multiple doses of 400 mg b.i.d.. At lower doses, detailed ECG analysis showed no changes in electrophysiology compared to placebo. Exposure-response modelling of the cardiac intervals revealed an exposure range of KH176 without effects on cardiac conduction and provided a threshold of 1000 ng/mL above which changes in intervals could occur. After single- and multiple-dose administration, the pharmacokinetics of KH176 was more than dose proportional. KH176 accumulated to a small extent and food only slightly affected the pharmacokinetics of KH176, which was considered clinically irrelevant. Renal excretion of unchanged KH176 and its metabolite represents a minor pathway in the elimination of KH176. As expected in healthy volunteers no effects on redox biomarkers were observed.

**Conclusion:**

The study deemed that KH176 is well tolerated up to single doses of 800 mg and multiple doses of 400 mg b.i.d. and has a pharmacokinetic profile supportive for a twice daily dosing. Only at high doses, KH176 causes clinically relevant changes in cardiac electrophysiology, including prolonged QTc interval and changes in T wave morphology. A Phase 2 clinical trial (100 mg b.i.d., orally) has been conducted recently of which the final results are expected Q1 2018.

**Trial registration:**

NCT02544217. Registered. ISRCTN43372293. Retrospectively registered.

**Electronic supplementary material:**

The online version of this article (10.1186/s13023-017-0715-0) contains supplementary material, which is available to authorized users.

## Background

Over 1150 genes have been identified to encode for proteins located in the mitochondria [1]. Mutations in these genes, like those involved in oxidative phosphorylation, can cause mitochondrial disease. This genetic heterogeneity is one of the numerous factors explaining the phenotypic variability seen in mitochondrial diseases, including many mono-, and multisystem phenotypes in childhood and adulthood [2]. Although the rate of deterioration is variable, the disease course is often progressive and the prognosis of some of the childhood diseases is even very poor [3]. With a minimal prevalence rate of about 1 in 4300–5000 [4], there is a clear need for therapy for these devastating disorders.

Currently, there is no effective treatment for most mitochondrial disorders and the care for these patients is mainly supportive [4]. Many treatment strategies have been attempted, including gene therapy, exercise and nutritional therapy, modulation of cell signalling and the manipulation of reactive oxygen species [5–7]. The latter has been subject of many studies and clinical trials [8, 9], since it has been recognised that cell redox imbalance play a key role in the pathogenesis of many of the clinical manifestations of mitochondrial diseases [5, 10–12].

KH176 (and its active metabolite KH176m) is a potent intracellular redox-modulating agent targeting these reactive oxygen species. Preclinical studies in patient-derived fibroblasts with a wide variety of gene defects associated with mitochondrial dysfunction show that KH176 was able to protect these cells against a toxic perturbation of their redox balance. Additionally, a 4 week- in vivo study showed that KH176 was able to significantly improve motor performance and gait in *Ndufs4* ^*−/−*^ mice [13]. Translating preclinical results to an anticipated human dose indicates that 100 mg b.i.d. is expected to be within a putatively efficacious range.

This study aims to test the safety, tolerability and pharmacokinetics of KH176 in human subjects after oral administration of a single dose (SD) as well as multiple doses (MD), to obtain a safe and putatively efficacious dosing regimen of KH176 for a next Proof of Concept study.

## Methods

**Fig. 1:**
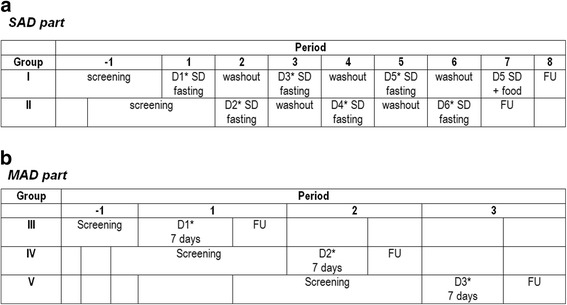
Study design. **a**. SAD study. D = Dose; SD = Single dose; FU = follow-up. * Each dose was followed by an interim safety and 24-h PK evaluation, which determined the dose selection for the next dose. A washout period of at least 4 days had to be taken into account. **b**. MAD study. Gr = group; D = Dose; FU = follow-up * Each dose was followed by an interim 7-days safety and PK evaluation, which determined the dose selection for the next dose

The compound as described herein was tested in a double-blinded, randomized, placebo-controlled, single-centre study in healthy male volunteers (Fig. 1). The single ascending dose (SAD) part has a partial alternating crossover design (*n* = 6) and the multiple-ascending dose (MAD) part has a sequential group design. Randomization was 2:1 (2 active for each placebo). All subjects received placebo once.

### Study population

For both the SAD and MAD study, healthy men between 18 and 55 years of age with a body mass index (BMI) of 18.0–30.0 kg/m^2^ were recruited. Good physical and mental health was established by medical history, physical examination, electrocardiogram (ECG) and vital signs recording, and results of clinical chemistry, hematology and urinalysis testing within 4 weeks prior to the first dose. Participants agreed to stay in the clinic during the first 24 h after dosing (SAD) and during Day 8 (MAD) and refrain from multivitamins and dietary supplements and grapefruit juice at least 14 Days prior to the first dosing, from alcohol 7 Days prior to the first dosing, from strenuous exercise, beverages containing quinine and from xanthine-derivates (e.g. caffeine) 48 h prior to the clinical admission and during the study. Only non-smokers (at least 3 months) were eligible for inclusion. Exclusion criteria included: clinically significant allergies, positive serology for hepatitis B surface antigen, hepatitis C antibodies, HIV1 of HIV2, history of alcohol or drug abuse in the past 2 years, history of cancer, surgery or active illness of the gastro-intestinal tract that might interfere with absorption, intake of any enzyme-affecting drugs in the 30 days prior to the first dosing period, use of any medication, herbal medicine or dietary supplement from 14 days prior to the first dosing (except for occasional paracetamol intake), participation in a trial of an investigational product in the 2 months prior to the first dosing, blood donation in the 2 months prior to the first dosing, history of hypersensitivity or idiosyncrasy to any of the components of the investigational drug, positive drug, alcohol or cotinine test at screening or admission, clinically relevant abnormal laboratory findings, ECG recordings, vital signs or physical or mental findings at screening, and/or major surgery and/or prolonged immobilisation (more than 2 weeks) within the 3 months prior to the screening.

### Study drug

KH176 (or ((S)-6-hydroxy-2,5,7,8-tetramethyl-N-((R)-piperidin-3-yl)chroman-2-carboxamide hydrochloride; Patent WO2014011047 A1) was available as a powder for reconstitution with saline. Placebo was a NaCl salt/bitrex powder for reconstitution with saline.

### Study design

The trial had a double-blinded, randomized, placebo-controlled, single-centre design. For the SAD part, a partial alternating crossover design and for the MAD part a sequential group design was applied.

In the SAD part the effects of 6 single orally administered ascending doses of KH176 or placebo were investigated alternately dosed to two groups of 6 healthy male subjects (4 active; 2 placebo per group). Dose escalation to the next dose level was done after evaluation of the safety and the first 24-h pharmacokinetic results of the previous dose. The single dose part included a food effect investigation; the 100-mg dose was administered following the intake of a high calorie/high fat breakfast to the same subjects as who received this dose in fasting conditions. In the MAD study 3 multiple ascending doses of KH176 were administered for 7 Days to 3 sequential groups of 6 healthy male subjects each (4 active; 2 placebo per group).

### Formulation and administration

KH176 (solution) was administered orally. Single doses of 10, 30, 100, 300, 800 and 2000 mg were administered in the SAD part. Multiple oral doses of 100, 200 and 400 mg were administered b.i.d. for 7 Days in the MAD part. The starting dose for the SAD was ~150 fold lower than the no observable adverse effect level (NOAEL) of both dogs and rats. Anticipated exposures at this starting dose were just below the Minimum Anticipated Biological Effect Level (MABEL). Placebo (an in taste and appearance matching oral liquid) was also administered orally on one occasion in the single-ascending dose part and b.i.d. for 7 Days in the MAD part.

### Safety assessment

Safety was assessed using standard vital signs, clinical laboratory results for blood chemistry, haematology and urinalysis, continuous cardiac telemetry and a 12-lead ECG at 1, 2, 4, 6, 8, 12 and 24 h post dosing. The changes from baseline (pre-dose assessment on Day 1) for body weight, physical examinations, vital signs, ECG-variables and clinical laboratory variables were determined for each time point. Treatment-emergent laboratory and ECG abnormalities as well as adverse events were monitored throughout the study, up to 28 Days after the last study drug intake.

Since preclinical studies in rats demonstrated the occurrence of phospholipidosis, the presence of phospholipidosis was determined by the concentration of di-docosahexaenoyl (22:6)-bis(monoacylglycerol) phosphate (di-22:6-BMP) in a midstream urine sample and by electron microscopy of peripheral leucocytes at Day 1 and 7 in the MAD study [14].

### Pharmacokinetic analysis

Plasma samples for pharmacokinetic analyses were taken pre-dose and at 0.5, 1, 1.5, 2, 3, 6, 8, 12, and 24 h after dosing (SAD) and pre-dose at Day 1, 2, 4, 7, and post-dose at Day 1 and Day 7 at 0.5, 1, 1.5, 2, 3, 4, 6, 8, 12 h after dosing (MAD). All samples were stored at <−70 °C until analysis. Quantification of KH176 and its metabolite KH176m were performed using a validated LC-MS/MS method with good (max 15–20% CV) selectivity, precision and accuracy, little carry over effect, good stability of both the solutions and the samples.

For mean value calculations, all values below the limit of quantification (LOQ) were set to zero. If <50% of the values at a given time point were below the LOQ (BLQ), these values were set to zero for calculation of the mean value. If >50% of the values at a given time point were BLQ, no mean value was calculated. The non-compartmental pharmacokinetic analysis was performed using Phoenix, Version 6.3 (Pharsight Corporation, Mountain View, CA, USA). Plasma concentration-time profiles of KH176 and its metabolite KH176m were determined for the SAD and MAD part and for trough concentrations in the MAD part. The PK parameters were calculated on the basis of the actual blood sampling time points relative to dosing. Selected pharmacokinetic parameters (C_max_, t_max_, t_1/2_, AUC_last_ and AUC_0-inf_) following single-dose administration and selected pharmacokinetic parameters C_max_, t_max_, t_1/2_, AUC_tau_, the accumulation factor (R_acc_) and time to reach steady state) following multiple dose administration were determined. In urine, the percentage of dose excreted in urine was determined for the single- and multiple-dose administration.

### Pharmacodynamic analysis

Blood samples for pharmacodynamic analyses were taken pre-dose and at 3, 6 and 24 h post dosing at Day 7 (MAD). All samples were stored in −80 °C until analysis. Quantification of oxidized and reduced glutathione was performed at York Bioanalytical solutions using a previously described validated method [15]. Change from baseline in the concentrations of oxidized and reduced glutathione (GSH/GSSG) was calculated.

### ECG post-hoc analysis

During the interim evaluations at dose escalation, machine-read ECGs and telemetry in the clinic indicated a prolongation of the corrected QT interval by KH176, as indicated by QTcB, the Bazett corrected QT interval, particularly strong at high dosages of the compound. Correction of the QT interval is indicated to allow comparison between QT values over different heart rates, since the QT interval shortens with higher heart rates. In the post-hoc analysis, we have used the Fridericia formula (QTcF = QT/RR^1/3^), which is more reliable compared to the QTcB outside the range of 60–100 bpm. The goal of this post-hoc optimization study was to repeat the machine-read ECG assessment with a highly automated computer-assisted approach where in addition to the re-evaluation of standard intervals (PR, QRS and QT intervals), a set of parameters describing repolarization morphology were considered. The morphology indices were the TpTe interval (interval from the T wave apex to the end of the T wave), the TpTe/QT index (the ratio between the TpTe interval and the QT interval), Tamp (the amplitude in microvolt units of the T wave) and TSym (an index of repolarization morphology based on the symmetry of the T wave).

ECGs were digitally recorded using a Schiller AT104 ECG machine (500 Hz, 1 μV). Cardiac interval measurements were performed on the Global Superimposed Median Beat (GSMB), a methodology that allows measurements that consider each of the 12 individual median beats [16]. This measurement methodology ensures that the PR, QRS, and QT interval are measured from the earliest onset in any lead to the latest deflection in any lead. Cardiologist overview of the computer-based measurements is based on the superimposed (overlapped) display of the individual median beats, to assure measurements are performed at the earliest onset of any viable lead to the latest offset of any viable lead.

The RR interval used for heart-rate correction of the QT interval (QTcB) was based on all the beats in the ten second recording.

Other cardiac parameters were computed from the 12-lead vector magnitude VM (e.g. the square root of the sum of squares at each digital sample) computed from the individual median beats. On the VM lead, the apex of the T wave is placed and used to compute the TpTe interval (using the end of the T wave from the GSMB) and the T wave amplitude (height in microvolt of the VM T peak from the isoelectric line). Additional file 1: Figure S1A shows an example with all the calipers involved: the GSMB leads are drawn in black and the VM lead is drawn in green. T wave symmetry index was computed using a proprietary approach based on Gaussian Mesa function modeling (GMF) of the repolarization waves [17]. Briefly, the ascending and descending phases of the VM T wave are modeled by two independent half-Gaussian curves (see Additional file 1: Figure S1). The standard deviations of these functions (σ1 and σ2) and indicators of the ascending/descending speed and their ratio is an index of symmetry (TSym = σ1/σ2; TSym = 1 for a perfectly symmetric T wave, TSym <1 for slow-ascending/fast-descending T waves, *T* > 1 for fast-ascending/slow-decending T waves).

For the pre-dose (baseline) ECGs, all parameters per time point were calculated as the averages of the triplicate ECGs. Computation of QTcB and QTcF was performed using the RR interval averaged from the total ECG acquisition duration (10 s) and in the case of the triplicate ECGs is based on the average QT and average HR of the replicate ECGs.

The over-reading cardiologist provided a clinical interpretation for each ECG at each time point. Each ECG was classified as Normal, Abnormal Clinically Insignificant (ACI), or Abnormal Clinically Significant (ACS), based on absolute changes in QTc interval (> 450 ms), changes from baseline (>60 ms) and the presence of secondary arrhythmias. Results of this post-hoc analysis were used for an exposure-response evaluation for the changes in the electrophysiological parameters as a function of the concentration of KH176.

### Ethics

All studies were conducted at the Drug Research Unit Ghent in accordance with the Declaration of Helsinki and the Good Clinical Practice guidelines established by the International Conference on Harmonization (ICH). An independent medical ethical committee approved the protocol (University Hospital of Ghent). All participants have signed informed consent prior to their enrolment.

This trial has been registered at clinicaltrials.gov prior to the conduction of the study (NCT02544217) and retrospectively at 23–06-2017 (ISRCTN43372293).

### Statistical analysis

The tolerability and safety data were compared between KH176 and placebo using descriptive summary statistics. Pharmacokinetic and pharmacodynamic parameters were analyzed by treatment group on the per-protocol set and summarized using descriptive statistics. Dose proportionality of log transformed C_max_ and AUC values was explored graphically. The effect of food on the pharmacokinetics was explored by calculating the geometric means and 90% confidence interval of the ratio (fed/fasted) for AUC and C_max_. The pharmacodynamic endpoints were analyzed descriptively by treatment group on the per-protocol set as a change from baseline. Pharmacokinetic-effect modelling of PK/ECG data was explored visually and by an exposure-response evaluation for the changes in the electrophysiological parameters as a function of the concentration of KH176.

## Results

### Study population

For the SAD part of the study, 14 healthy male subjects divided in two groups of 7 subjects were included. Two subjects prematurely withdrew from the study after dosing period 2 for non-medical reasons. For the MAD part of the study, 18 healthy male subjects divided in 3 groups of 6 subjects were included. Two subjects prematurely withdrew from the study for non-medical reasons and were replaced. There were no protocol violations in either part of the study that excluded subjects from the analysis set(s) and, therefore, all subjects included in this study were evaluable for pharmacokinetics, pharmacodynamics, safety, and tolerability. For demographics, see Table 1.

**Table 1:** Summary of demographic characteristics for subjects included in the SAD and MAD study

	SAD		MAD			
	Group I (*N* = 7)^a^	Group II (*N* = 7)^a^	Group III 100 mg BID (*N* = 4)	Group IV 200 mg BID (*N* = 4)	Group V 400 mg BID (*N* = 4)	Placebo (*N* = 6)
Age (years)						
Mean (SD)	30.4 (10.5)	32.0 (11.3)	40.8 (13.0)	34.5 (11.6)	44.0 (8.2)	44.7 (9.8)
Median	28.0	31.0	44.0	35.0	43.0	48.5
Range	(22–52)	(18–45)	(24–51)	(23–45)	(37–53)	(28–54)
Height (cm) at screening						
Mean (SD)	178.07 (6.56)	176.01 (8.11)	184.63 (2.81)	181.75 (5.25)	175.70 (3.18)	175.55 (8.25)
Median	177.50	177.00	184.90	183.75	176.70	171.60
Range	(165.7–184.5)	(163.0–187.5)	(181.5–187.2)	(174.0–185.5)	(171.1–178.3)	(170.4–191.5)
Weight (kg) at screening						
Mean (SD)	82.86 (10.56)	74.29 (12.61)	89.05 (11.84)	77.85 (11.96)	81.10 (4.82)	72.03 (6.06)
Median	80.00	78.60	89.30	80.30	82.80	72.30
Range	(69.8–100.0)	(55.2–88.6)	(77.2–100.4)	(61.2–89.6)	(74.2–84.6)	(62.6–78.8)
BMI (kg/m^2^) at screening						
Mean (SD)	26.09 (2.51)	24.01 (4.01)	26.10 (2.74)	23.60 (3.82)	26.28 (1.67)	23.43 (1.98)
Median	26.10	22.80	26.25	24.90	26.70	23.50
Range	(22.5–29.4)	(19.0–29.6)	(23.1–28.8)	(18.2–26.4)	(23.9–27.8)	(21.2–26.6)
Race						
White	7 (100.0%)	7 (100.0%)	4 (100.0%)	4 (100.0%)	4 (100.0%)	6 (100.0%)

^a^6 subjects were enrolled of whom 1 withdrew and has been replaced

### Safety and tolerability

Following administration of a single dose of KH176 to subjects in the fasted state (SAD study), a total of 43 adverse events (AEs) were reported by 14 subjects (56%; Additional file 2: Table S1A). The majority of AEs (35) was rated as mild in severity whereas there were 6 moderate and 2 severe AEs. Up to and including the 800 mg dose, no relationship between the presence of placebo or KH176, the dose of KH176 and the number or the severity of reported AEs could be discerned (Additional file 2: Table S1). However, 28 of the 43 reported AEs were reported by subjects in the highest dose group of which 6 and 2 were of moderate and severe severity, respectively. The 2 severe AEs were nausea and headache. Following administration of placebo, a total of 7 AEs were reported by 3 subjects (25%). Of note, 4 of these AEs were rated as moderate in severity. Headache was the most frequently reported AE with a total of 7 subjects including 2 placebo subjects. The majority of AEs occurred incidentally, i.e., was reported by only 1 or 2 subjects. Adverse events reported by more than 2 subjects and not by placebo subjects were psychiatric symptoms, dizziness, oral paraesthesia and prolonged QT at the electrocardiogram and telemetry. All these events occurred in the highest dose group (2000 mg dose) and were reported by 3 subjects each.

Following administration of a multiple doses of KH176 (MAD study), a total of 29 AEs were reported by 10 subjects (83.3%; Additional file 2: Table S1B). The majority of AEs (27) was rated as mild in severity whereas there were 2 moderate AEs, one each in the 200- and 400-mg dose groups. No relationship to dose and the number of reported AEs or severity could be discerned. Headache was the most frequently reported AE with a total of 11 subjects including 5 placebo subjects reporting this AE. Except for skin irritation, all AEs occurred incidentally, i.e., were reported by only 1 or 2 subjects in the KH176 treated subjects. Skin irritation was also reported by 2 placebo subjects and, therefore, no AE was reported by more than 2 KH176-treated subjects and not by placebo subjects.

The level of di-22:6-BMP was variable at baseline. There was no increase in di-22:6-BMP for the treatment groups versus placebo (change from Day 1 to Day 7 0.74–2.50; −1.44–1.32 and −0.69-1.46 ng/mg creatinine for group III, IV and V respectively and −0.92-4.31 ng/mg creatinine for placebo). No increased prevalence of the presence of phospholipidosis in either granulocytes or monocytes as evaluated by electron microscopy was present in the KH176-treated group versus the placebo-treated group.

### Pharmacokinetic analysis

#### SAD study

**Fig. 2:**
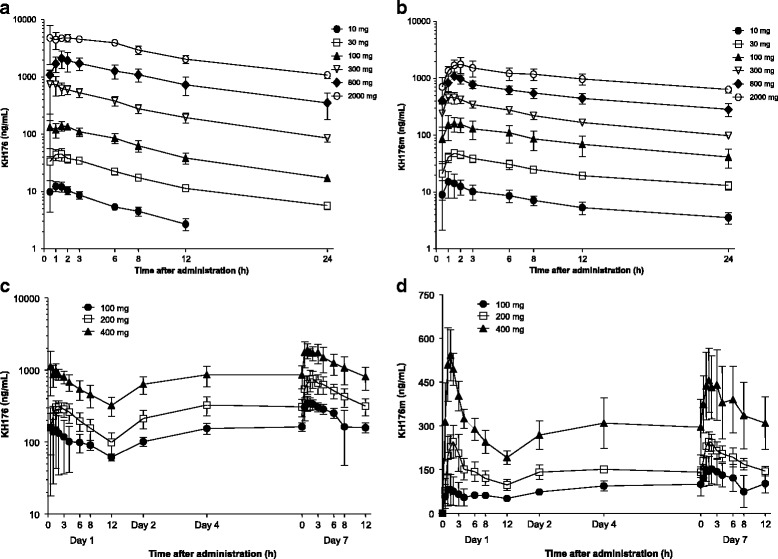
Mean plasma concentrations of KH176 and its metabolite KH176m after single and multiple dose administration to healthy subjects. **a**. Plasma-concentration/time curve of KH176 for the SAD study (fasted state). **b**. Plasma-concentration/time curve of KH176m for the SAD study (fasted state). **c**. Plasma-concentration/time curve of KH176 for the MAD study. **d**. Plasma-concentration/time curve of KH176m for the MAD study. *Loglinear scale*

The plasma concentrations-time profiles of KH176 (Fig. 2a) showed that the median t_max_ was between 0.75 and 1.5 h after administration and varied little with dose. Thereafter, the KH176 concentrations decreased in a biphasic way. The t_1/2_ was approximately 10 h and varied little among dose groups (Table 2). The elimination phase following administration of a dose of 10 mg was not well characterized and, therefore, t_1/2_ could not be estimated for this dose only.

**Table 2:** Summary of plasma pharmacokinetic variables of KH176

A. For the SAD study									
			SAD study						
Dose			10 mg (*N* = 4)	30 mg (*N* = 4)	100 mg (*N* = 4)	100 mg (*N* = 4)	300 mg (*N* = 4)	800 mg (*N* = 4)	2000 mg (*N* = 4)
Food status			fasted	fasted	fasted	fed	fasted	fasted	fasted
C_max_	(ng/mL)	Geomean	12.9	56.2	167	165	766	2170	5990
		CV% geomean	21.3	21.0	27.2	7.40	53.9	27.9	20.9
t_max_	(h)	Geomean	1.25	1.25	1.00	2.50	0.992	1.50	0.750
		CV% geomean	(0.500–1.50)	(0.500–1.50)	(0.500–2.00)	(2.00–3.00)	(0.500–3.00)	(1.00–1.50)	(0.500–3.00)
AUC_last_	(h*ng/mL)	Geomean	75.0	389	1310	1650	6320	21,000	61,200
		CV% geomean	14.8	8.16	16.7	1.67	20.2	27.5	9.07
AUC_0-inf_	(h*ng/mL)	Geomean		474	1540	1970	7500	25,800	79,100
		CV% geomean		8.39	13.1	1.04	19.1	33.9	8.82
t_1/2_	(h)	Geomean	NA	10.3	9.10	9.09	9.64	9.80	11.5
		CV% geomean		14.6	15.2	3.83	4.29	19.1	4.73
B. For the MAD study									
			MAD study						
Dose			100 mg b.i.d (*n* = 4)		200 mg b.i.d (*n* = 4)		400 mg b.i.d (*n* = 4)		
Day			1	7	1	7	1	7	
C_max_	(ng/mL)	Geomean	184	353	313	748	1330	2100	
		CV% geomean	57.0	19.1	20.0	29.3	27.3	27.2	
t_max_*	(h)	Geomean	1.25	1.00	1.75	2.00	1.00	1.50	
		CV% geomean	(0.500–8.00)	(0.500–1.50)	(1.50–2.00)	(1.50–2.00)	(0.500–1.50)	(0.500–3.00)	
AUC_tau_	(h*ng/mL)	Geomean	1090	2760	2250	5960	6890	14,900	
		CV% geomean	49.3	22.6	26.1	26.8	19.2	28.2	
Racc		Geomean		2.52		2.65		2.17	
		CV% geomean		31.2		2.5		14.4	

*Geomean* geometric mean, *h* hour, *NA* not assessable, *R* accumulation ratio, *median (range)

The shape of the plasma concentration-time profiles of KH176m (Fig. 2b) resembled that of the parent compound but concentrations were lower. C_max_ was reached at the same time or slightly later when compared to KH176 (Additional file 2: Table S2). Thereafter, KH176m plasma concentrations decreased in a biphasic way and the elimination was characterized by a t_1/2_ of approximately 16 h without any notable effect of dose.

In the presence of food, the absorption of KH176 was slower as indicated by a median t_max_ that shifted from approximately 1 h in fasted condition to 2.5 h in fed condition for both analytes (Table 2). Exposure under fed conditions in terms of AUC_0-inf_ increased slightly for KH176 whereas that to KH176m decreased slightly (AUC_0-inf_ 1.28 (90%CI 1.12–1.45) for KH176 and 0.90 (90%CI 0.57–1.41) for KH176m). The t_1/2_ was not affected by food.

**Fig. 3:**
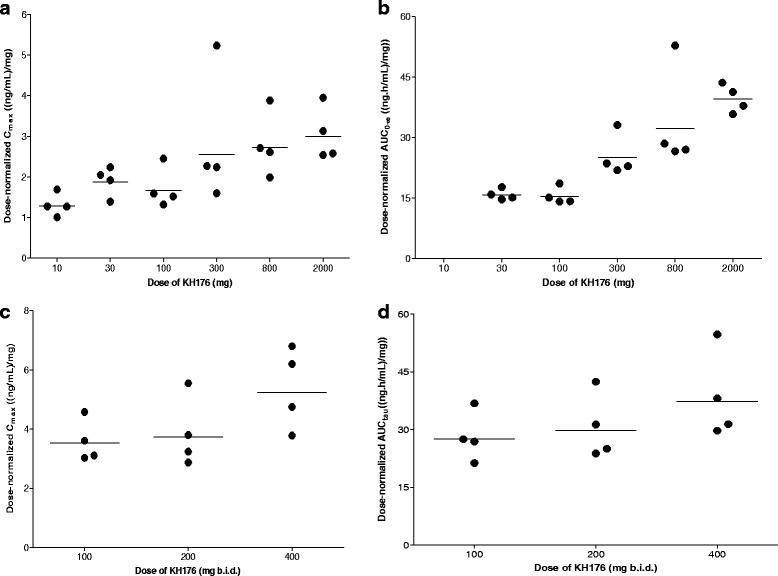
Dose-normalized individual values for C_max_ and AUC_0-inf_ of KH176 for the SAD and the MAD study. **a**. Dose normalized C_max_ for the SAD study. **b**. Dose normalized AUC_0-inf_ for the SAD study. **c**. Dose normalized C_max_ for the MAD study. **d**. Dose normalized AUC_0-inf_ for the MAD study. *The horizontal lines depict the geometric mean value*

A graphical exploration for dose-proportionality of the pharmacokinetics of KH176 indicated that with increasing single dose there was a more than proportional increase in C_max_ and AUC_0-inf_ (Fig. 3).

Regardless of the dose and when combining KH176 and KH176m, approximately 16% of the administered dose was excreted in urine. Unchanged KH176 accounted for approximately 12%.

#### MAD study

Visual inspection of the mean trough concentration-time curves indicated that steady concentrations of KH176 were reached by Day 4 of dosing (of note: Day 4 was the first time point of measurement of trough values; Fig. 2c).

The plasma concentration-time profiles of KH176 after single- and multiple-dose administration were similar. Peak concentrations were attained between 1 and 2 h after drug administration (Table 2). Following attainment of C_max_, the KH176 plasma concentrations declined rapidly. KH176 accumulated as shown by values for the accumulation index between doses varying from 2.17 to 2.65.

After multiple dosing, the shape of the plasma concentration-time profiles of KH176m resembled that of parent compound but concentrations were lower (Fig. 2d). On Day 7, C_max_ was reached at approximately the same time when compared to KH176. After multiple dosing, the accumulation of the metabolite KH176m was less pronounced when compared to that of KH176 as indicated by values for the accumulation index varying from 1.19 to 1.86.

A graphical exploration for dose-proportionality of the pharmacokinetics of KH176 after multiple dosing indicated that with increasing multiple doses there was a more than proportional increase in C_max_ and AUC_tau_, which was most pronounced at the 400 mg dose (Fig. 3).

On Day 7 and when combining KH176 and KH176m, the %dose excreted in urine varied from 18.0 to 25.1% between doses. Unchanged KH176 accounted for 14.0 to 18.1%.

### Pharmacodynamic analysis

No significant alterations in the GSH/GSSG ratio were observed.

### Posthoc analysis QTc prolongation

A QTcF prolongation was present after single-dose administration of 800 and 2000 mg KH176 (see Additional file 3: Figure S3 for a representative example). The largest median baseline increase observed was 46.8 ms (1 h post-dose), whereas the largest individual change was 64.7 ms (2000 mg group; Additional file 4: Figure S2). This QTcF prolongation was associated with moderate but clear changes of morphology, namely a reduction of the T wave amplitude, a prolongation of the TpTe interval (in both absolute terms and relatively to the QT interval), and the symmetry (shape) of the T wave. At lower doses, KH176 does not seem to affect repolarization. Changes were also observed on other cardiac intervals: the QRS interval increased but only at a dose of 2000 mg, whereas the PR interval increased progressively also at lower doses of KH176.

The MAD part of the study showed the same effects, particularly for the 400 mg dose group (Additional file 4: Figure S2). A dose of 100 mg b.i.d. had no effect on QTcF and the time curve for this dose was indistinguishable from placebo. On Day 4 and later, administration of 200 mg b.i.d. increased QTcF and a median maximum change from baseline was observed on Day 5 of 13 msec. A further increase in QTcF was observed with a dose of 400 mg b.i.d. and the median maximum change from baseline at trough of 29 msec was observed on Day 6.

Clinically relevant changes were observed in at the peak concentrations in 2 individuals in the 2000 mg subgroup and did not include appearance of notched and/or bumps on any of the T-waves (all leads).

**Fig. 4:**
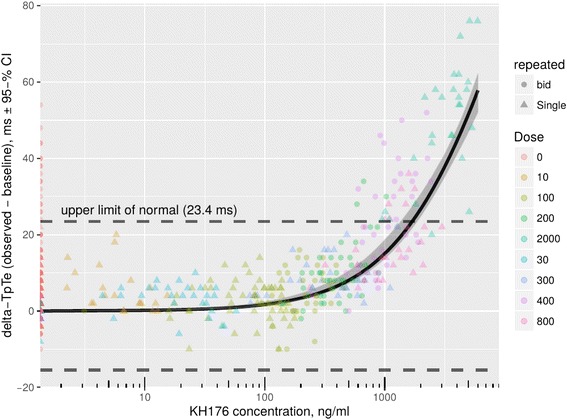
Exposure-response analysis of KH176 plasma concentrations and a change from baseline for ECG derived TpTe intervals. The upper limit of normal (23.4 ms) is derived from the 95% confidence interval of the pre-dose values. In healthy subjects a dose of 100 mg BID resulted in maximum concentrations ranging from 303 to 458 ng/mL

When the change in the TpTe interval is correlated to the KH176-exposure, a clear dose-dependency is observed (Fig. 4). In exposures lower than 500 ng/ml, no significant prolongation of the TpTe interval compared to placebo was observed. When increasing the dose and the plasma concentration, the TpTe interval increases. When the highest (non-tolerated) dose was administered (2000 mg), all subjects have a prolonged TpTe interval.

## Discussion

Mitochondrial disorders are a devastating group of disorders for which there is an urgent need for treatment development. In preclinical studies, KH176 showed promising properties in ameliorating the viability and phenotype of cells and mice affected by mitochondrial disease. The objective of this study was to evaluate the tolerability, safety, pharmacokinetics and pharmacodynamics of single- and multiple-ascending doses of KH176 in healthy male subjects.

KH176 was well tolerated in doses up to 800 mg SD and 400 mg b.i.d.. Headache was the most frequently reported AE in both the KH176 - and the placebo treated groups. Although there was no clear dose-response relationship for any of the adverse effects after single and multiple dose administration, unforeseen markedly more severe adverse events were reported after a single dose of 2000 mg. At this dose, which is a factor 10 above the anticipated daily human efficacious dose of 200 mg/day, nausea, vomiting, dizziness and psychiatric disturbances were reported, along with a prolonged corrected QT time.

The shape of the plasma concentration-time profiles of KH176 and its metabolite KH176m were similar after single- and multiple-dose administration. The pharmacokinetics of KH176 showed several aspects. In particular, the pharmacodynamics of KH176 were characterized by a i) median t_max_ between 0.75 and 2.0 h, ii) terminal t_1/2_ of about 10 h, iii) biphasic elimination, and a iv) more than dose proportional increases in both C_max_ and AUC.

In vitro, KH176 is metabolized by CYP3A4 and excreted by the PgP efflux pump. We did not observe signs for auto-induction in this study such as a decline in pre-dose concentrations. Without wishing to be bound by any theory, the more than proportional increase with dose could be caused by saturation of the PgP efflux pump for which KH176 is a substrate, as evidenced by the fact that half-life and clearance doesn’t change with dose, but rather the availability seems to change with dose. Up to 25.1% of an administered dose is excreted via urine after multiple dosing. In steady state conditions at 100 mg BID dosing the maximum concentration reached ranges within 303–458 ng/mL for KH176 and 89.4–204 ng/mL for KH167m (hence the maximum concentrations of these active moieties ranged within 392.4–662 ng/mL).

Based on the results of the multiple-dose administration, steady state was reached at the first time point of measurement of trough concentrations (Day 4). Based on the estimated t_1/2_, it is expected that steady state will be reached after 2 to 3 Days of dosing. KH176 was well tolerated in the presence of food, and the tolerability and safety profile in the presence and absence of food was similar. Although the point estimates (as well as the sample covariance) for both C_max_ and AUC_0-inf_ did not completely remain within the usually accepted range of 0.8 to 1.25 (Table 2), no special measures are warranted regarding the intake of KH176.

KH176 clearly modifies cardiac repolarisation in a dose-dependent manner. QTcF prolongation was present after single-dose administration of 800 and 2000 mg and after multiple doses of 400 mg of KH176 b.i.d.. Post-hoc studies showed that this QTcF prolongation was associated with changes in morphology and other cardiac intervals. Detailed analysis of the ECGs during the single-dose administration of 200, 100, 30 and 10 mg and multiple oral doses of 100 and 200 mg b.i.d. showed no cardiac electrophysiological abnormalities. Moreover, the changes in the QTcF interval in the 800 mg KH176 single dose and the 400 mg b.i.d KH176 dose did not reach the threshold for clinical relevance. The KH176 related changes in cardiac repolarisation include a reduction of T wave amplitude, a prolongation of the TpTe interval and a reduction of the T wave symmetry index. The reduction in T wave amplitude is largely explained by changes in heart rate and also seen in the placebo group. The prolongation of the TpTe interval and the reduction on the symmetry index indicate a prolongation of the descending phase of the T wave. With pure hERG blockade, there is a direct relationship between increasing plasma drug concentrations and the risk for torsades. Importantly, although the specific T wave morphology changes observed in this study have been associated with the hERG potassium channel blockade [18], the IC_50_ of hERG blocking for KH176 was 3 times higher than the C_max_ for the highest dose.

The clinical relevance of these changes in cardiac electrophysiology are not clear yet. Notably, there were 2 subjects with a potentially clinically relevant change (>60 ms) at the peak concentration after administration of 2000 mg but no signs of arrhythmias or severe morphologic changes (such as T wave bumps, notches, etc) were observed. One should be cautious when translating these healthy volunteer results to a patient population. Since the sensitivity to torsades de pointes also depends on many other factors including cardiovascular disease, alcoholic liver disease, obesity, hypertension, and/or electrolyte disturbances [19, 20], high concentrations (e.g. more than a total daily dose of 1000 mg) of KH176 preferably should not be given to patients with any of these risk factors. Moreover, until dose adjustments for patients with concomitant medication (also metabolized by CYP3A4) are determined, high concentrations (e.g. more than a total daily dose of 1000 mg) of KH176 should preferably not be given to these patients either since unpredictable pharmacokinetics will possibly lead to plasma concentrations of KH176 above the currently defined safety threshold.

Since the mechanism of KH176 is based on correction of an abnormal redox balance, as expected, we did not observe any pharmacodynamic changes in the healthy male volunteers.

Comparing human exposure to mouse exposure in the in vivo mouse studies and benchmarking human exposure to in vitro activity of KH176 and metabolites indicates that 100 mg b.i.d. dosing results in a putatively efficacious exposure to be tested in the following Proof of Concept study.

## Conclusion

We conclude that administration of single doses up to and including 800 mg or multiple doses up to 400 mg b.i.d. for 7 days of KH176, a new small redox-modulating molecule developed to treat mitochondrial(−related) diseases and conditions, is safe and well tolerated in healthy male volunteers. Although doses above the anticipated human efficacious dose could lead to prolongation of the QTc interval with T-wave abnormalities, these changes did not reach the threshold for clinical relevance in these men. We have recently performed a phase 2 study in m.3243A > G carriers, administering the anticipated efficacious dose which did not lead to changes in cardiac electrophysiology in healthy men (100 mg KH176 b.i.d). Based on the study reported here, patients with cardiac abnormalities were excluded for safety reasons.

## Additional files

Additional file 1: Figure S1. Posthoc ECG assessment methodology. A. Example of an ECG adapted on the Global Superimposed Median Beat (GSMB). B. T wave symmetry index was computed by modeling the T wave in two independent half-Gaussian curves. The standard deviations of these functions (σ1 and σ2) and indicators of the ascending/descending speed. (PDF 143 kb)

Additional file 2: Table S1. Summary of treatment-emergent adverse events by system organ class and preferred term. **Table S2.** Summary of plasma pharmacokinetic variables of KH176m. **Table S3.** Largest median an largest individual increase in the ECG parameters for the SAD and the MAD study. (DOCX 36 kb)

Additional file 3: Figure S3. Representative example of the changes in the intervals in an individual in the 2000 mg group. (PNG 63 kb)

Additional file 4: Figure S2. Posthoc ECG assessment results. A. Change in QTcF (median increase from baseline; SAD study). B. Change in TpTe (median increase from baseline; SAD study). C. Change in the T-wave symmetry index (median increase from baseline; SAD study). D. Change in QTcF (median increase from baseline; SAD study). E. Change in TpTe (median increase from baseline; MAD study). F. Change in the T-wave symmetry index (median increase from baseline; MAD study). (PDF 341 kb)

## Abbreviations

ACI: Abnormal clinically insignificant
ACS: Abnormal clinically significant
AE: Adverse events
b.i.d.: bis in die (two times daily)
BLQ: Below limit of quantification
GSMB: Global superimposed median beat
HR: Heart rate
LOQ: Limit of quantification
MABEL: Minimum anticipated biological effect level
MAD: Multiple ascending dose
MD: Multiple dose
NOAEL: No observed adverse effects level
QTcB: Bazett’s corrected QT interval
QTcF: Fridericia’s corrected QT interval
SAD: Single ascending dose
SD: Single dose
Tamp: the amplitude in microvolt units of the T wave
TEAE: Treatment-emergent adverse events
TpTe interval: interval from the T wave apex to the end of the T wave
TpTe/QT index: the ratio between the TpTe interval and the QT interval
TSym: index of repolarization morphology based on the symmetry of the T wave
