# Supplementary figures and images for: KH176 under development for rare mitochondrial disease: a first in man randomized controlled clinical trial in healthy male volunteers
Source: Orphanet J Rare Dis. 2017 Oct 16;12:163. doi: 10.1186/s13023-017-0715-0 (PMC5644106; doi:10.1186/s13023-017-0715-0)

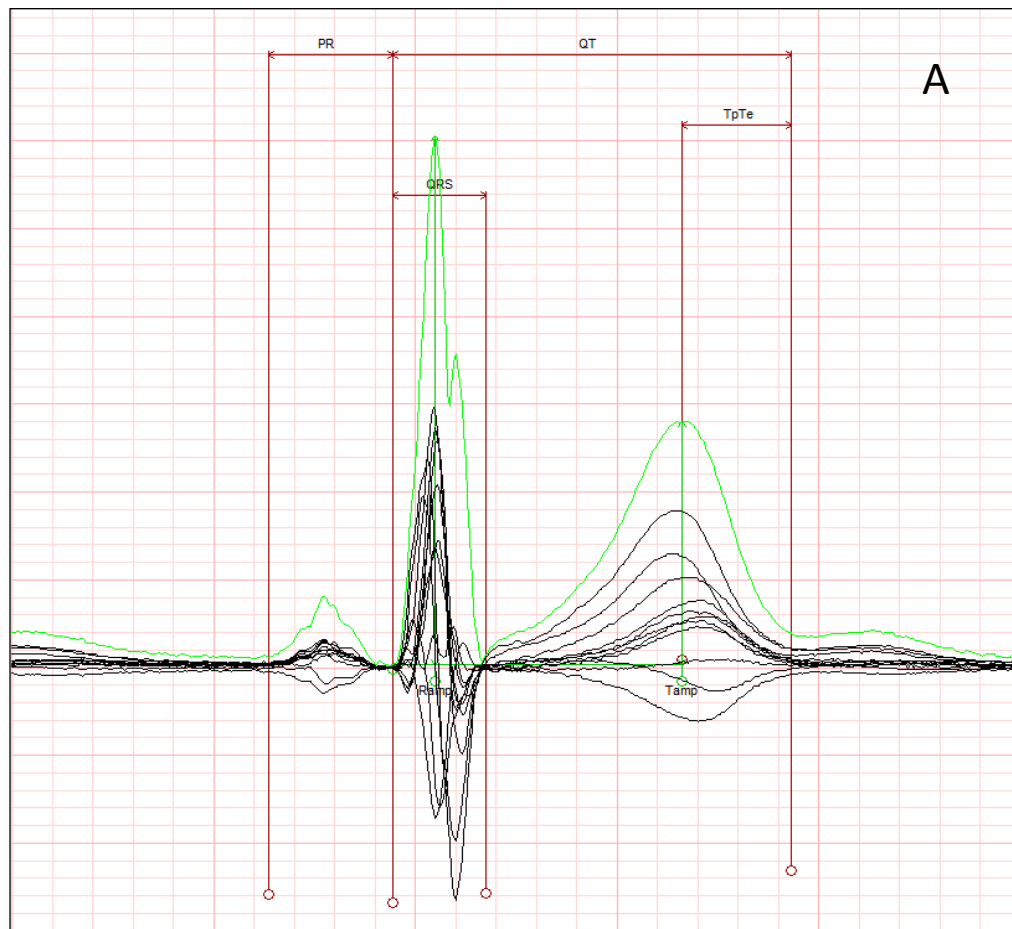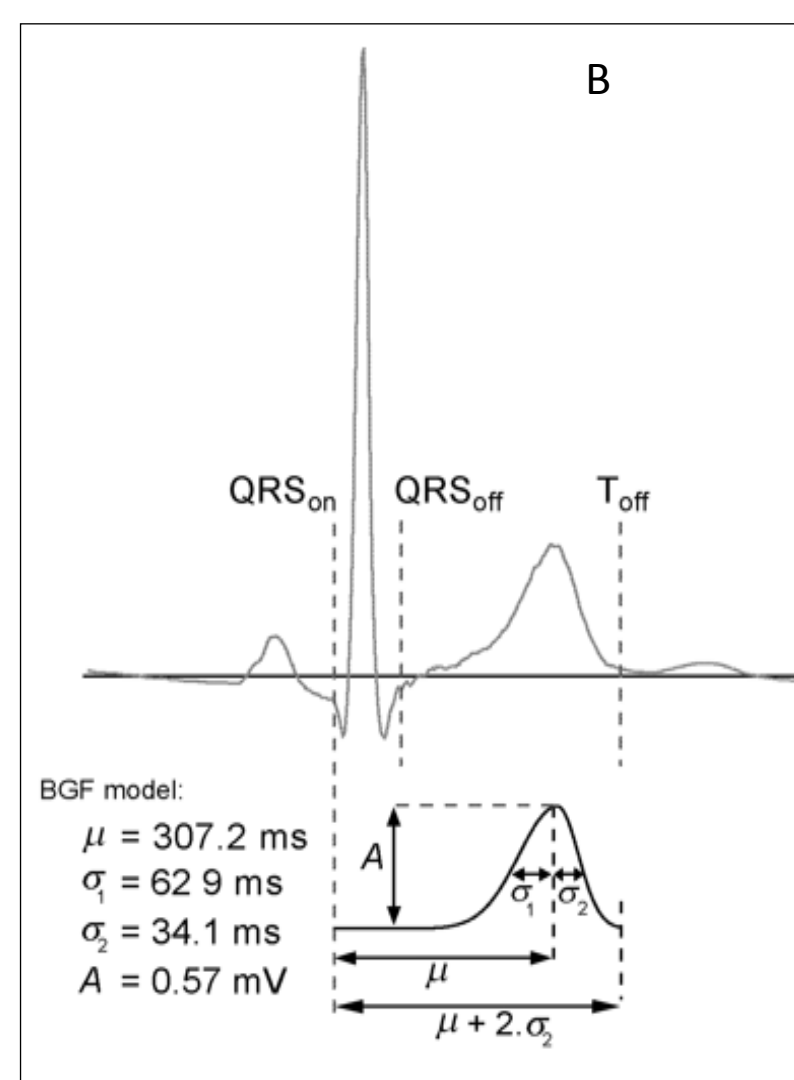

Supplement: Supplementary file 1 — Posthoc ECG assessment methodology. A. Example of an ECG adapted on the Global Superimposed Median Beat (GSMB). B. T wave symmetry index was computed by modeling the T wave in two independent half-Gaussian curves. The standard deviations of these functions (σ1 and σ2) and indicators of the ascending/descending speed. (PDF 143 kb) [file 13023_2017_715_MOESM1_ESM.pdf]

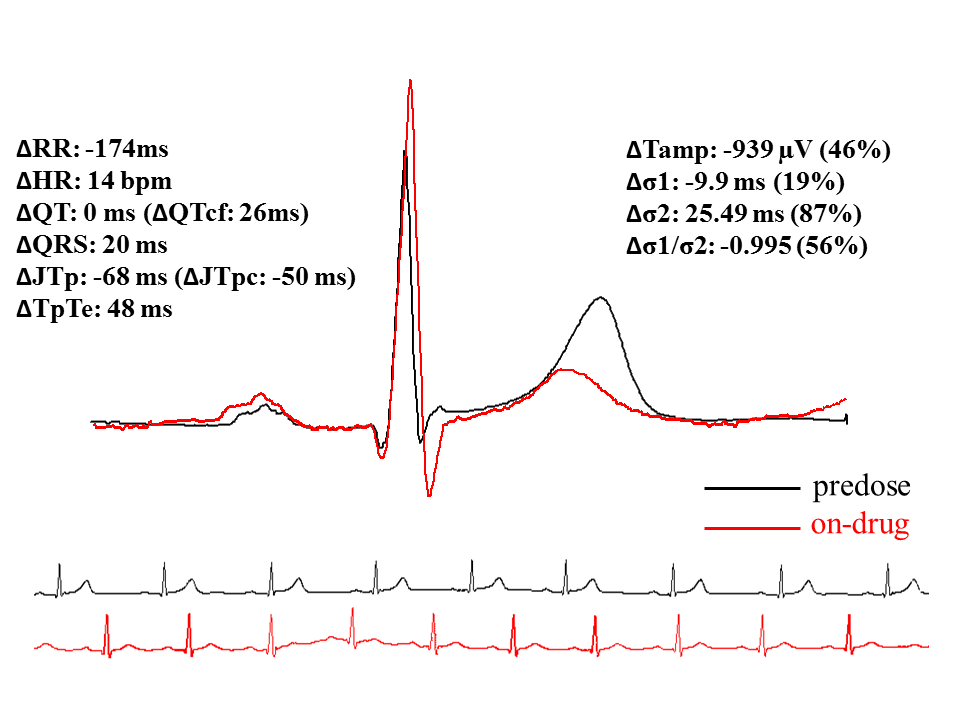

Supplement: Supplementary file 3 — Representative example of the changes in the intervals in an individual in the 2000 mg group. (PNG 63 kb) [file 13023_2017_715_MOESM3_ESM.png]
