# Supplementary material for: KH176 under development for rare mitochondrial disease: a first in man randomized controlled clinical trial in healthy male volunteers
Source: Orphanet J Rare Dis. 2017 Oct 16;12:163. doi: 10.1186/s13023-017-0715-0 (PMC5644106; doi:10.1186/s13023-017-0715-0)
Supplement: Supplementary file 2 — Summary of treatment-emergent adverse events by system organ class and preferred term. Table S2. Summary of plasma pharmacokinetic variables of KH176m. Table S3. Largest median an largest individual increase in the ECG parameters for the SAD and the MAD study. (DOCX 36 kb) [file 13023_2017_715_MOESM2_ESM.docx]

**Additional files**

**Table S1. Summary of treatment-emergent adverse events by system organ class and preferred term**

A. For SAD study

| **System organ class** | **Group I Placebo (N=6)** | **Group II Placebo (N=6)** | **Group I 10 mg (N=4)** | **Group II 30 mg (N=4)** | **Group I 100 mg (N=4)** | **Group II 300 mg (N=4)** | **Group I 800 mg (N=4)** | **Group II 2000 mg (N=4)** | **Group I Placebo + Food (N=2)** | **Group I 100 mg + Food (N=4)** |
| --- | --- | --- | --- | --- | --- | --- | --- | --- | --- | --- |
| **Preferred term** | **n (%)** | **n (%)** | **n (%)** | **n (%)** | **n (%)** | **n (%)** | **n (%)** | **n (%)** | **n (%)** | **n (%)** |
| Any TEAE | 2 (33.3%) | 1 (16.7%) | 2 (50.0%) | 3 (75.0%) | 4 (100.0%) | 1 (25.0%) | 0 | 4 (100.0%) | 1 (50.0%) | 1 (25.0%) |
| Blood and lymphatic system disorders | 0 | 0 | 0 | 1 (25.0%) | 0 | 0 | 0 | 0 | 0 | 0 |
| Lymphadenopathy | 0 | 0 | 0 | 1 (25.0%) | 0 | 0 | 0 | 0 | 0 | 0 |
| Psychiatric disorders | 0 | 0 | 0 | 0 | 0 | 0 | 0 | 3 (75.0%) | 0 | 0 |
| Bradyphrenia | 0 | 0 | 0 | 0 | 0 | 0 | 0 | 1 (25.0%) | 0 | 0 |
| Depersonalisation | 0 | 0 | 0 | 0 | 0 | 0 | 0 | 1 (25.0%) | 0 | 0 |
| Hallucination, visual | 0 | 0 | 0 | 0 | 0 | 0 | 0 | 1 (25.0%) | 0 | 0 |
| Nervous system disorders | 2 (33.3%) | 0 | 0 | 2 (50.0%) | 3 (75.0%) | 1 (25.0%) | 0 | 3 (75.0%) | 0 | 0 |
| Dizziness | 0 | 0 | 0 | 0 | 0 | 0 | 0 | 3 (75.0%) | 0 | 0 |
| Dysgeusia | 0 | 0 | 0 | 0 | 0 | 1 (25.0%) | 0 | 0 | 0 | 0 |
| Headache | 2 (33.3%) | 0 | 0 | 1 (25.0%) | 3 (75.0%) | 0 | 0 | 1 (25.0%) | 0 | 0 |
| Presyncope | 0 | 0 | 0 | 1 (25.0%) | 0 | 0 | 0 | 0 | 0 | 0 |
| Cardiac Disorders | 0 | 0 | 0 | 0 | 0 | 0 | 0 | 2 (50.0%) | 0 | 0 |
| Bundle branch block right | 0 | 0 | 0 | 0 | 0 | 0 | 0 | 2 (50.0%) | 0 | 0 |
| Respiratory, thoracic and mediastinal disorders | 0 | 0 | 1 (25.0%) | 0 | 1 (25.0%) | 0 | 0 | 0 | 0 | 0 |
| Oropharyngeal discomfort | 0 | 0 | 0 | 0 | 1 (25.0%) | 0 | 0 | 0 | 0 | 0 |
| Rhinorrhoea | 0 | 0 | 1 (25.0%) | 0 | 1 (25.0%) | 0 | 0 | 0 | 0 | 0 |
| Gastrointestinal disorders | 1 (16.7%) | 1 (16.7%) | 0 | 1 (25.0%) | 1 (25.0%) | 0 | 0 | 4 (100.0%) | 0 | 0 |
| Abdominal pain | 0 | 1 (16.7%) | 0 | 0 | 1 (25.0%) | 0 | 0 | 0 | 0 | 0 |
| Diarrhoea | 0 | 1 (16.7%) | 0 | 0 | 0 | 0 | 0 | 0 | 0 | 0 |
| Nausea | 1 (16.7%) | 0 | 0 | 0 | 0 | 0 | 0 | 2 (50.0%) | 0 | 0 |
| Odynophagia | 0 | 0 | 0 | 1 (25.0%) | 0 | 0 | 0 | 0 | 0 | 0 |
| Paraesthesia oral | 0 | 0 | 0 | 0 | 0 | 0 | 0 | 3 (75.0%) | 0 | 0 |
| Retching | 0 | 0 | 0 | 0 | 0 | 0 | 0 | 1 (25.0%) | 0 | 0 |
| Vomiting | 0 | 0 | 0 | 0 | 0 | 0 | 0 | 2 (50.0%) | 0 | 0 |
| Musculoskeletal and connective tissue disorders | 1 (16.7%) | 0 | 1 (25.0%) | 1 (25.0%) | 0 | 0 | 0 | 0 | 0 | 0 |
| Arthralgia | 0 | 0 | 0 | 1 (25.0%) | 0 | 0 | 0 | 0 | 0 | 0 |
| Musculoskeletal stiffness | 0 | 0 | 1 (25.0%) | 0 | 0 | 0 | 0 | 0 | 0 | 0 |
| Pain in extremity | 1 (16.7%) | 0 | 0 | 0 | 0 | 0 | 0 | 0 | 0 | 0 |
| General disorders and administration site conditions | 1 (16.7%) | 0 | 0 | 0 | 1 (25.0%) | 0 | 0 | 2 (50.0%) | 0 | 1 (25.0%) |
| Catheter site pain | 0 | 0 | 0 | 0 | 0 | 0 | 0 | 0 | 0 | 1 (25.0%) |
| Chills | 0 | 0 | 0 | 0 | 0 | 0 | 0 | 1 (25.0%) | 0 | 0 |
| Fatigue | 0 | 0 | 0 | 0 | 1 (25.0%) | 0 | 0 | 0 | 0 | 0 |
| Influenza like illness | 1 (16.7%) | 0 | 0 | 0 | 0 | 0 | 0 | 0 | 0 | 0 |
| Malaise | 0 | 0 | 0 | 0 | 0 | 0 | 0 | 1 (25.0%) | 0 | 0 |
| Investigations | 0 | 0 | 0 | 0 | 0 | 0 | 0 | 3 (75.0%) | 1 (50.0%) | 0 |
| Blood pressure diastolic increased | 0 | 0 | 0 | 0 | 0 | 0 | 0 | 1 (25.0%) | 0 | 0 |
| Blood pressure systolic increased | 0 | 0 | 0 | 0 | 0 | 0 | 0 | 1 (25.0%) | 0 | 0 |
| Electrocardiogram (uncorrected) QT prolonged | 0 | 0 | 0 | 0 | 0 | 0 | 0 | 3 (75.0%) | 0 | 0 |
| Eosinophil count increased | 0 | 0 | 0 | 0 | 0 | 0 | 0 | 0 | 1 (50.0%) | 0 |

*n = number of subjects; TEAE = Treatment-Emergent Adverse Event*

B. For MAD study

|  | **Group III 100 mg BID (N=4)** | **Group IV 200 mg BID (N=4)** | **Group V 400 mg BID (N=4)** | **Placebo (N=6)** |
| --- | --- | --- | --- | --- |
|  | **n (%)** | **n (%)** | **n (%)** | **n (%)** |
| Any TEAE | 3 (75.0%) | 3 (75.0%) | 4 (100.0%) | 5 (83.3%) |
| Infections and infestations | 1 (25.0%) | 0 | 0 | 0 |
| Nasopharyngitis | 1 (25.0%) | 0 | 0 | 0 |
| Psychiatric disorders | 0 | 0 | 1 (25.0%) | 0 |
| Nightmare | 0 | 0 | 1 (25.0%) | 0 |
| Nervous system disorders | 2 (50.0%) | 2 (50.0%) | 1 (25.0%) | 3 (50.0%) |
| Dizziness | 0 | 0 | 0 | 1 (16.7%) |
| Head discomfort | 0 | 1 (25.0%) | 0 | 1 (16.7%) |
| Headache | 2 (50.0%) | 1 (25.0%) | 1 (25.0%) | 3 (50.0%) |
| Eye disorders | 1 (25.0%) | 1 (25.0%) | 0 | 0 |
| Conjunctival haemorrhage | 1 (25.0%) | 0 | 0 | 0 |
| Eye irritation | 1 (25.0%) | 0 | 0 | 0 |
| Vision blurred | 0 | 1 (25.0%) | 0 | 0 |
| Gastrointestinal disorders | 1 (25.0%) | 0 | 2 (50.0%) | 2 (33.3%) |
| Abdominal pain | 1 (25.0%) | 0 | 0 | 0 |
| Diarrhoea | 0 | 0 | 1 (25.0%) | 0 |
| Nausea | 0 | 0 | 1 (25.0%) | 2 (33.3%) |
| Skin and subcutaneous tissue disorders | 0 | 1 (25.0%) | 2 (50.0%) | 2 (33.3%) |
| Rash macular | 0 | 1 (25.0%) | 0 | 0 |
| Skin irritation | 0 | 1 (25.0%) | 2 (50.0%) | 2 (33.3%) |
| Renal and urinary disorders | 0 | 0 | 1 (25.0%) | 0 |
| Polyuria | 0 | 0 | 1 (25.0%) | 0 |
| General disorders and administration site conditions | 1 (25.0%) | 0 | 0 | 1 (16.7%) |
| Feeling cold | 0 | 0 | 0 | 1 (16.7%) |
| Feeling hot | 1 (25.0%) | 0 | 0 | 0 |
| Investigations | 0 | 0 | 4 (100.0%) | 0 |
| Blood creatine kinase increased | 0 | 0 | 1 (25.0%) | 0 |
| Electrocardiogram (uncorrected) QT prolonged | 0 | 0 | 1 (25.0%) | 0 |
| Lipase increased | 0 | 0 | 2 (50.0%) | 0 |
| Injury, poisoning and procedural complications | 0 | 0 | 1 (25.0%) | 1 (16.7%) |
| Wound | 0 | 0 | 1 (25.0%) | 1 (16.7%) |

*n = number of subjects; TEAE = Treatment-Emergent Adverse Event*

**Table S2. Summary of plasma pharmacokinetic variables of KH176m**

A. For the SAD study

|  |  |  | **SAD study** | | | | | | |
| --- | --- | --- | --- | --- | --- | --- | --- | --- | --- |
| Dose |  |  | **10 mg (N = 4)** | **30 mg (N = 4)** | **100 mg (N = 4)** | **100 mg (N = 4)** | **300 mg (N = 4)** | **800 mg (N = 4)** | **2000 mg (N = 4)** |
| Food status |  |  | fasted | fasted | fasted | fed | fasted | fasted | fasted |
| **C_max_** | **(ng/mL)** | Geomean | 14.2 | 49.4 | 168 | 117 | 497 | 1100 | 1780 |
|  |  | CV% geomean | 49.5 | 9.00 | 29.6 | 20.0 | 17.6 | 9.58 | 26.3 |
| **t**_max_* | **(h)** | Geomean | 1.25 | 1.50 | 1.25 | 2.50 | 1.24 | 1.50 | 1.75 |
|  |  | CV% geomean | (1.00-2.00) | (1.00-2.00) | (1.00-2.00) | (2.00-3.00) | (1.00-2.00) | (1.50-2.00) | (1.00-2.00) |
| **AUC**_last_ | **(h*ng/mL)** | Geomean | 151 | 547 | 1810 | 1590 | 4830 | 11700 | 23900 |
|  |  | CV% geomean | 26.7 | 12.4 | 37.4 | 26.1 | 11.8 | 20.2 | 22.2 |
| **AUC_0-inf_** | **(h*ng/mL)** | Geomean | 234 | 881 | 2690 | 2410 | 6890 | 18700 | 41300 |
|  |  | CV% geomean | 26.7 | 19.6 | 38.4 | 29.1 | 11.0 | 26.7 | 10.5 |
| **t**_1/2_ | **(h)** | Geomean | 16.6 | 17.8 | 15.4 | 14.9 | 14.3 | 17.2 | 18.9 |
|  |  | CV% geomean | 5.86 | 19.0 | 8.44 | 7.86 | 18.9 | 18.5 | 18.7 |

*Geomean = geometric mean; h = hour; R = accumulation ratio; *median (range)*

B. For the MAD study

|  |  |  | **MAD study** | | | | | |
| --- | --- | --- | --- | --- | --- | --- | --- | --- |
| Dose |  |  | 100 mg b.i.d (n = 4) | | 200 mg b.i.d (n = 4) | | 400 mg b.i.d (n = 4) | |
| Day |  |  | 1 | 7 | 1 | 7 | 1 | 7 |
| **C_max_** | **(ng/mL)** | Geomean | 99.4 | 152 | 251 | 250 | 550 | 450 |
|  |  | CV% geomean | 20.9 | 37.5 | 18.0 | 10.6 | 14.1 | 28.3 |
| **t_max_*** | **(h)** | Geomean | 1.50 | 1.00 | 1.75 | 1.50 | 1.25 | 1.50 |
|  |  | CV% geomean | (1.00-8.00) | (1.00-2.03) | (1.00-2.00) | (1.00-2.00) | (1.00-1.50) | (1.00-3.00) |
| **AUC_tau_** | **(h*ng/mL)** | Geomean | 702 | 1310 | 1680 | 2220 | 3600 | 4270 |
|  |  | CV% geomean | 29.2 | 29.5 | 21.0 | 12.7 | 11.1 | 30.5 |
| **Racc** |  | Geomean |  | 1.86 |  | 1.32 |  | 1.19 |
|  |  | CV% geomean |  | 46.7 |  | 13.1 |  | 24.7 |

*Geomean = geometric mean; h = hour; R = accumulation ratio; *median (range)*

**Table S3.** Largest median an largest individual increase in the ECG parameters for the SAD and the MAD study

|  |  | SAD study | | | | MAD study | | | |
| --- | --- | --- | --- | --- | --- | --- | --- | --- | --- |
|  |  | 800 mg | | 2000 mg | | 200 mg | | 400 mg | |
|  |  | **Largest median baseline increase** | **Largest individual increase** | **Largest median baseline increase** | **Largest individual increase** | **Largest median baseline increase** | **Largest individual increase** | **Largest median baseline increase** | **Largest individual increase** |
| QTcF |  | 9 | 26 | 46.8 | 64.7 | 13.3 | 28.3 | 29.2 | 43.7 |
| HR | bpm |  |  | 10.3 | 23.3 |  |  |  |  |
| PR interval | ms | 10.3 | 19.3 | 15.3 | 20.7 | 12 |  | 19 |  |
| QRS interval | ms | 5.3 | 8 | 25.7 | 30 |  |  | 9.7 | 15.3 |
| TpTe interval | ms | 21.7 | 36.7 | 58.7 | 76 | 24 | 46 | 42.3 | 62.7 |
| T-wave amplitude | MV | 600 | 706 | 734 | 994 |  |  |  |  |
| T-wave symmetry index |  | 0.42 | 0.85 | 0.87 | 1.1 | 0.45 | 0.6 | 0.63 | 0.99 |
