# Supplementary material for: KH176 under development for rare mitochondrial disease: a first in man randomized controlled clinical trial in healthy male volunteers
Source: Orphanet J Rare Dis. 2017 Oct 16;12:163. doi: 10.1186/s13023-017-0715-0 (PMC5644106; doi:10.1186/s13023-017-0715-0)
Supplement: Supplementary file 4 — Posthoc ECG assessment results. A. Change in QTcF (median increase from baseline; SAD study). B. Change in TpTe (median increase from baseline; SAD study). C. Change in the T-wave symmetry index (median increase from baseline; SAD study). D. Change in QTcF (median increase from baseline; SAD study). E. Change in TpTe (median increase from baseline; MAD study). F. Change in the T-wave symmetry index (median increase from baseline; MAD study). (PDF 341 kb) [file 13023_2017_715_MOESM4_ESM.pdf]

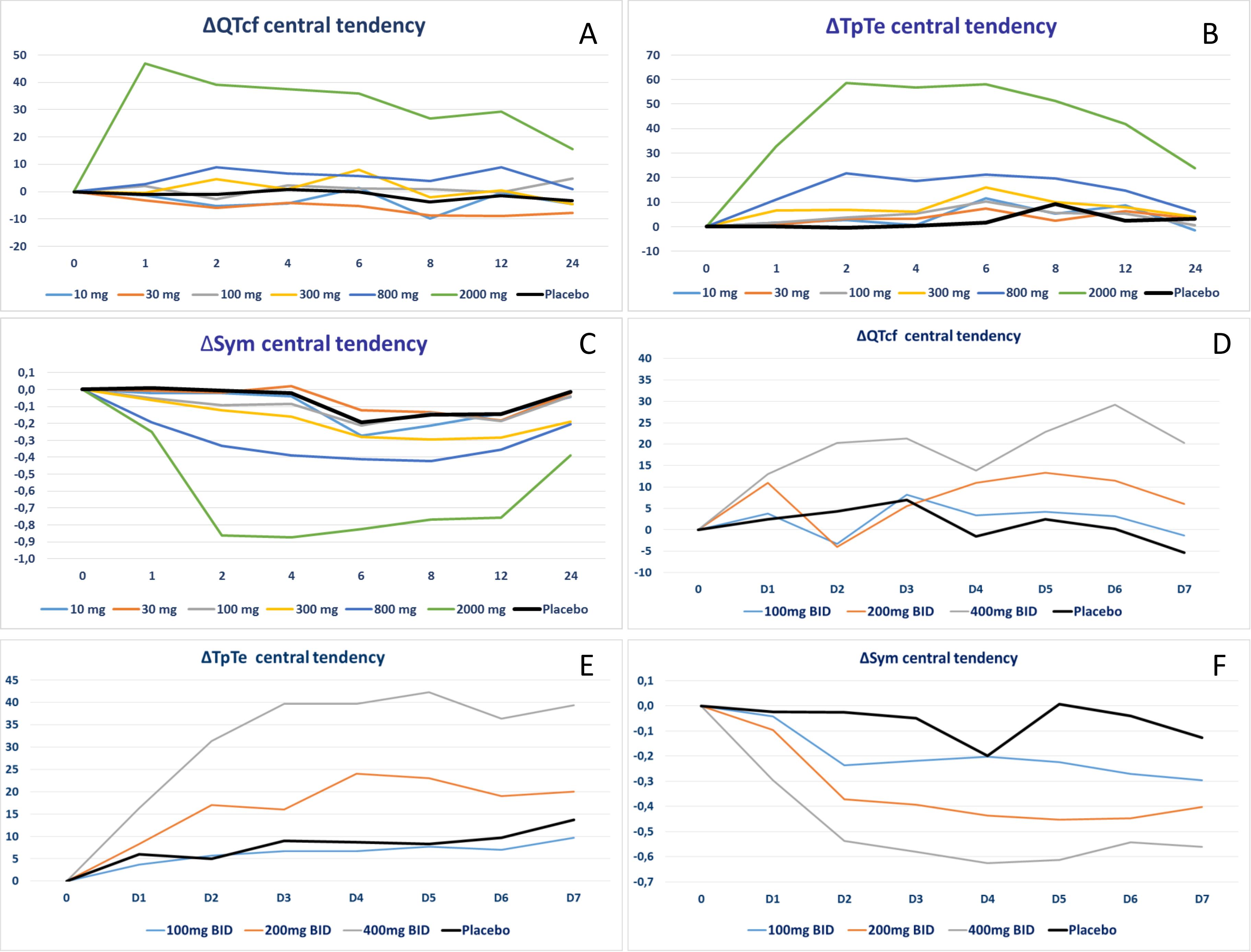

ΔQTcf central tendency
A
50
40
30
20
10
0
-10
-20
0 1 2 4 6 8 12 24
10 mg 30 mg 100 mg 300 mg 800 mg 2000 mg Placebo
ΔTpTe central tendency
B
70
60
50
40
30
20
10
0
-10
0 1 2 4 6 8 12 24
10 mg 30 mg 100 mg 300 mg 800 mg 2000 mg Placebo
ΔSym central tendency
C
0,1
0,0
-0,1
-0,2
-0,3
-0,4
-0,5
-0,6
-0,7
-0,8
-0,9
-1,0
0 1 2 4 6 8 12 24
10 mg 30 mg 100 mg 300 mg 800 mg 2000 mg Placebo
ΔQTcf central tendency
D
40
35
30
25
20
15
10
5
0
-5
-10
0 D1 D2 D3 D4 D5 D6 D7
100mg BID 200mg BID 400mg BID Placebo
ΔTpTe central tendency
E
45
40
35
30
25
20
15
10
5
0
0 D1 D2 D3 D4 D5 D6 D7
100mg BID 200mg BID 400mg BID Placebo
ΔSym central tendency
F
0,1
0,0
-0,1
-0,2
-0,3
-0,4
-0,5
-0,6
-0,7
0 D1 D2 D3 D4 D5 D6 D7
100mg BID 200mg BID 400mg BID Placebo
